# The association between cardiovascular diseases and their subcategories with the severity of chronic obstructive pulmonary disease: a large cross-sectional study based on a Chinese hospital population cohort

**DOI:** 10.3389/fcvm.2025.1502205

**Published:** 2025-02-13

**Authors:** Tianye Li, Lefu Chen, Hao Xu, Yanhong Zheng, Heying Yang, Hongjun Zhao, Chengshui Chen

**Affiliations:** ^1^ Key Laboratory of Interventional Pulmonology of Zhejiang Province, Department of Pulmonary and Critical Care Medicine, The First Affiliated Hospital of Wenzhou Medical University, Wenzhou, China; ^2^ Department of Internal Medicine, Nassau University Medical Center, East Meadow, NY, United States; ^3^ Zhejiang Province Engineering Research Center for Endoscope Instruments and Technology Development, Department of Pulmonary and Critical Care Medicine, Quzhou People’s Hospital, The Quzhou Affiliated Hospital of Wenzhou Medical University, Quzhou, China

**Keywords:** chronic obstructive pulmonary disease, cardiovascular disease, hypertension, coronary heart disease, severity

## Abstract

**Background:**

Current evidence suggests that cardiovascular disease (CVD) plays a role in the progression of chronic obstructive pulmonary disease (COPD). However, the relationship between CVD and the severity of COPD remains inadequately understood. Therefore, this study aims to elucidate the association between CVD and the severity of COPD.

**Methods:**

In this cross-sectional study involving 7,152 individuals with COPD., Logistic regression, subgroup and sensitivity analyses were employed to evaluate the association between CVD, its subcategories, and the severity of COPD.

**Results:**

Multivariable logistic regression analysis showed that CVD and hypertension remained independently associated with COPD severity (*P* < 0.001). Patients with CVD had a 1.701 times higher risk of developing severe or very severe COPD compared to those without CVD, while patients with hypertension had a 1.686 times higher risk of developing severe or very severe COPD compared to those without hypertension (*P* < 0.05). Subgroup analyses showed that the association between CVD and COPD severity remained stable among men, patients ≤ 70 years of age, patients > 70 years of age, BMI < 24 or ≥24 kg/m^2^, and never smokers, whereas coronary artery disease was significantly associated with COPD severity only among patients ≤ 70 years of age and never smokers (*P* < 0.05). In addition, hypertension was also stably associated with COPD severity among men, patients ≤ 70 years of age, patients > 70 years of age, BMI < 24 or ≥24 kg/m^2^, and never smokers. Sensitivity analyses reconfirmed the robustness of the associations of CVD and hypertension with COPD severity among patients who excluded bronchiectasis, tuberculosis, lung cancer, pulmonary hypertension, pulmonary heart disease, and diabetes (*P* < 0.05).

**Conclusion:**

The strong association between CVD and its subcategories (mainly hypertension) and the severity of COPD suggests that the potential risk of exacerbation of CVD should also be addressed in the clinical management of patients with COPD. However, limitations of the cross-sectional design may limit the extrapolation of the results, and more large prospective clinical cohort studies are needed in the future to further validate the association.

## Introduction

In recent years, chronic obstructive pulmonary disease (COPD) has continued to maintain a high mortality rate and remains a significant public health concern worldwide. It is predicted that by 2030, COPD will become the third leading cause of death globally (1, 2). Clinically, patients with COPD often experience recurrent symptom exacerbations, a progressive decline in lung function, reduced quality of life, and decreased physical activity, which can ultimately lead to fatal outcomes (3, 4). Cardiovascular diseases (CVD) are primarily caused by atherosclerosis, which is characterized by the narrowing or blockage of arteries due to plaque buildup from fatty deposits on the artery walls. There is growing evidence suggesting a strong co-existence of COPD and CVD, with an intricate relationship between the two (5). In addition to common risk factors like smoking and age, inflammation plays a critical role in connecting these diseases. In the progression of COPD, the significance of chronic inflammation cannot be ignored. Chronic inflammation inflicts damage on the lung parenchyma and surrounding airways, leading to persistent respiratory symptoms and irreversible airflow limitation (6). In addition, low-grade chronic inflammation is associated with increased rates of atherosclerosis and insulin resistance, which play an important role in the occurrence and development of CVD (7). Fianlly, the progression of COPD is influenced by several factors, particularly its comorbidities (8–10). Research has shown that CVD are major contributors to the exacerbation of chronic lung conditions and significantly increase the risk of mortality in individuals with COPD (11–13).

However, much of the existing research has focused on individual CVD and has often involved small sample sizes, lacking representation from the Chinese population. Therefore, to further investigate the relationship between CVD and the severity of COPD, a large population-based cohort of 40,000 participants from northwestern and southeastern China between 2014 and 2021 was selected for a cross-sectional study. According to the current research background, in order to fill some knowledge gaps, this study innovatively evaluate the association of CVD and its subclassification with the severity of COPD patients through multiple statistical methods including multivariate logistic regression analysis, subgroup analysis and sensitivity analysis, aiming to provide new clues and theoretical basis for clinical management and prognosis assessment of COPD patients.

## Methods

### Study population

**Figure 1:**
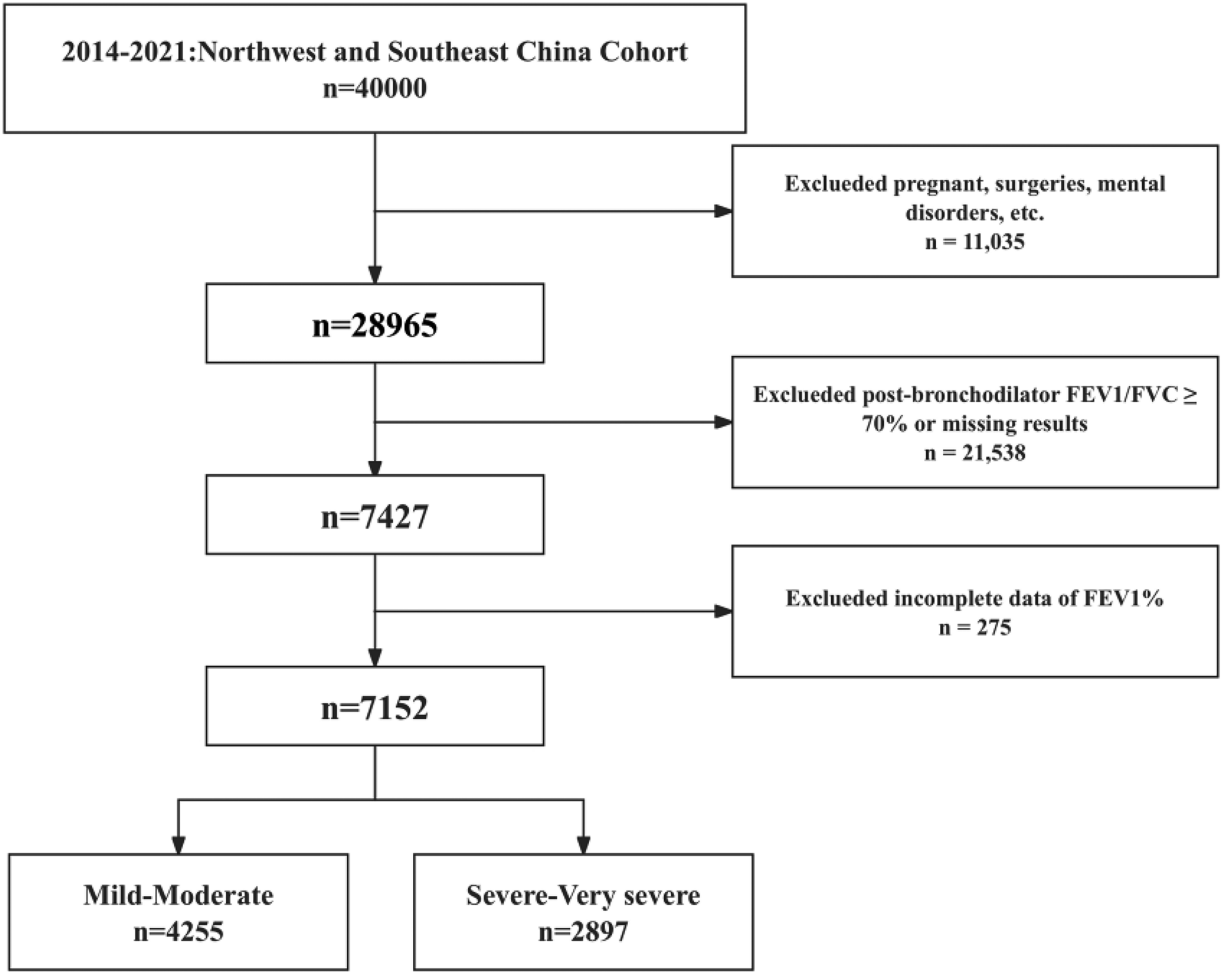
Study population inclusion flow chart. FEV1, forced expiratory volume in 1 s; FVC, forced vital capacity; FEV1%, percentage of predicted forced epiratory vlume in 1 s.

In this cross-sectional study, all participants were drawn from a population-based cohort in northwestern and southeastern China from 2014–2021. The cohort selected study participants from eight hospitals in five Chinese provinces with a total population of 40,000 through a multistage whole population sampling method. 7,152 individuals were included after excluding individuals with current psychiatric disorders, contraindications to pulmonary function testing, other conditions that affect pulmonary function test results, and those with significant missing information (Figure 1). The data for this study were derived from a multicenter cohort study, the protocols were reviewed and approved by all participating subcenters, all patients signed written informed consent, and the study complied with the basic principles of the Declaration of Helsinki.

### Assessment of CVD and its subcategories

In this study, CVD and its subcategories [hypertension, arrhythmia, and coronary heart disease (CHD)] were considered as exposure factors. Hypertension was defined as a history of previous hypertension, current use of antihypertensive medication, or systolic blood pressure (SBP)/diastolic blood pressure (DBP) ≥ 140/90 mmHg (14). Arrhythmia was defined as a history of previous arrhythmia or current use of antiarrhythmic medication, including atrial, supraventricular, or ventricular arrhythmias. CHD was defined as a history of previous CHD or the presence of typical CHD symptoms, signs, and imaging. Two groups were divided according to the prevalence of CVD and its subclassifications, respectively.

### COPD severity assessment

In this study, both COPD and its severity were determined by lung function. Following the GOLD criteria, COPD was defined as forced expiratory volume in the first second (FEV1)/forced vital capacity (FVC) < 70%, and this ratio was used to assess the degree of airway obstruction after a bronchodilator test (after inhalation of 400 *μ*g of salbutamol for at least 15 min) (15). A uniform spiral flow meter was used for all tests, and each subject underwent two pulmonary function tests, which were confirmed by repeating the test if the post-bronchodilator FEV1/FVC was less than 0.70 in both tests. Severity was graded primarily on the basis of FEV1 as a percentage of the predicted value: mild (GOLD 1): FEV1 ≥ 80% of predicted value. Moderate (GOLD 2): 50% ≤ FEV1 < 80% of predicted value. Severe (GOLD 3): 30% ≤ FEV1 < 50% expected. Very severe (GOLD 4): FEV1 < 30% predicted or FEV1 < 50% with chronic respiratory failure (16). In this study, participants were divided into two groups based on COPD severity: mild to moderate (*n* = 4,255) and severe to very severe (*n* = 2,897).

### Covariate collection

Data on participants' demographics, exposure factors, and medical conditions were gathered using questionnaires, spirometry, and blood pressure screenings. The main covariates used for this study included age, gender, body mass index (BMI), blood pressure, smoking, frequency of smoking, asthma, chronic bronchitis, bronchiectasis, emphysema, tuberculosis, lung cancer, pulmonary heart disease, chronic rhinitis, diabetes, BMI, SBP, DBP, and heart rate. BMI was defined as weight (kg)/height (m^2^), and SBP and DBP were the average of three consecutive measurements on different days. Smoking was defined as continuous smoking for more than 6 months, while smoking frequency was divided into three groups: never, occasional and daily. Chronic bronchitis was defined to include any of the following: (1) a previous diagnosis of chronic bronchitis; and (2) clinical symptoms of cough and sputum, with an onset lasting 3 months per year, for 2 or more consecutive years. Bronchiectasis was defined to include any of the following: (1) previous diagnosis of bronchiectasis; (2) imaging suggestive of bronchiectasis. Tuberculosis was defined to include any of the following: (1) previous diagnosis of tuberculosis; and (2) being on anti-tuberculosis medication. Lung cancer was defined as a history of physician-diagnosed lung cancer. Pulmonary heart disease was evaluated by a combination of self-reported past medical history and echocardiographic measurements during this hospitalization. Chronic rhinitis was diagnosed based on the patient's past medical history. Diabetes was defined as a history of a previous diagnosis of diabetes or being treated with glucose-lowering medication or having a fasting blood glucose ≥7.0 mmol/L or glycosylated haemoglobin ≥6.5% or a blood glucose ≥11.1 mmol/L after 2 h of an oral glucose tolerance test (17).

### Statistical analyses

All statistical analyses in this study were performed using SPSS 26.0. Continuous variables that conformed to normal distribution were expressed as mean ± standard deviation, and differences between groups were tested by independent samples *t*-test. Continuous variables that did not conform to normal distribution were described as median (quartiles) and differences between groups were tested using non-parametric tests. Categorical variables were described as frequencies (percentages), and differences between groups were tested using the chi-square test. Univariate logistic regression analysis was used to assess the association of all variables with COPD severity, and then variables with *P* < 0.05 were selected for multifactorial logistic regression analysis to assess the independent association of CVD including CHD, arrhythmia, and hypertension with COPD severity. Then, stratified associations of CVD and its subcategories with COPD severity were further assessed by constructing 8 subgroups based on gender (male or female), age (≤70 or >70 years), BMI (<24 or ≥24 kg/m^2^), and smoking frequency (never, occasional or daily). Finally, the independent association of CVD and its subcategories with COPD severity was further evaluated in sensitivity analysis by excluding variables that might have an important impact on COPD severity, such as bronchiectasis, tuberculosis, lung cancer, pulmonary heart disease, and diabetes. All tests were two-sided and were considered statistically significant at *P* < 0.05.

## Results

### Analysis of baseline information

As shown in Table 1, the study included 7,152 patients, of whom 5,846 (81.7%) were male, with a mean age of (68.36 ± 11.60) years. They were divided into two groups according to the severity of COPD: mild-moderate group and severe-very severe group. Compared with the mild-moderate group, the severe-very severe group was older, had a higher proportion of men and smokers, a higher proportion of occasional smokers, a higher prevalence of chronic bronchitis, emphysema, CVD, CHD and hypertension, but a lower prevalence of chronic rhinitis, and had higher levels of heart rate, but lower levels of BMI and FEV1/FVC (*P* < 0.05).

**Table 1:** Clinical characteristics of groups according to COPD severity.

	Overall	Mild-moderate	Severe-very severe	*P* value
*N*	7,152	4,255	2,897	
Age, years	68.36 ± 11.60	67.28 ± 12.03	70.00 ± 10.94	<0.001
Gender, *n* (%)				<0.001
Male	5,846 (81.7)	3,404 (80.0)	2,442 (84.3)	
Female	1,306 (18.3)	851 (20.0)	455 (15.7)	
Smoking, *n* (%)	717 (81.1)	373 (76.7)	344 (86.4)	<0.001
Smoking frequency, *n* (%)				0.026
Never smoker	6,912 (96.6)	4,102 (96.4)	2,810 (97.0)	
Occasional smoker	54 (0.8)	27 (0.6)	27 (0.9)	
Daily smoker	186 (2.6)	126 (3.0)	60 (2.1)	
Chronic bronchitis, *n* (%)	212 (3.0)	112 (2.6)	100 (3.5)	0.045
Bronchiectasis, *n* (%)	25 (0.3)	18 (0.4)	7 (0.2)	0.202
Emphysema, *n* (%)	228 (3.2)	108 (2.5)	120 (4.1)	<0.001
Tuberculosis, *n* (%)	33 (0.5)	16 (0.4)	17 (0.6)	0.197
Lung cancer, *n* (%)	6 (0.1)	3 (0.1)	3 (0.1)	0.692
Pulmonary heart disease, *n* (%)	9 (0.1)	3 (0.1)	6 (0.2)	0.171
Chronic rhinitis, *n* (%)	27 (0.4)	22 (0.5)	5 (0.2)	0.020
Diabetes, *n* (%)	44 (0.6)	23 (0.5)	21 (0.7)	0.327
CVD, *n* (%)	299 (4.2)	149 (3.5)	150 (5.2)	0.001
CHD, *n* (%)	50 (0.7)	22 (0.5)	28 (1.0)	0.025
Arrhythmia, *n* (%)	24 (0.3)	12 (0.3)	12 (0.4)	0.343
Hypertension, *n* (%)	364 (5.1)	189 (4.4)	175 (6.0)	0.003
BMI, kg/m^2^	22.76 ± 3.21	23.14 ± 3.09	22.21 ± 3.29	<0.001
SBP, mmHg	133.20 ± 19.63	134.84 ± 19.74	133.79 ± 18.10	0.545
DBP, mmHg	78.37 ± 10.87	78.99 ± 10.44	78.14 ± 11.76	0.402
Heart rate, bpm	78.83 ± 12.79	77.03 ± 11.83	80.86 ± 13.52	0.001
FEV1/FVC, %	55.80 ± 10.85	61.63 ± 7.02	47.25 ± 9.73	<0.001

COPD, chronic obstructive pulmonary disease; CVD, cardiovascular disease; CHD, coronary heart disease; BMI, body mass index; SBP, systolic blood pressure; DBP, diastolic blood pressure; FEV1, forced expiratory volume in the first second; FVC, forced vital capacity.

### Univariate and multivariate regression

Univariate logistic regression analysis showed that age, male, smoking, smoking frequency, chronic bronchitis, chronic rhinitis, emphysema, CVD, CHD, hypertension, BMI, heart rate, and FEV1/FVC were all associated with COPD severity (*P* < 0.05) (Table 2). Multivariate logistic regression analysis showed (Table 3), both CVD and hypertension were significantly associated with COPD severity in model 1 (*P* < 0.05). In model 2, a association between CVD, CHD, and hypertension with COPD severity was observed (*P* < 0.05). In the fully adjusted model 3, CVD and hypertension remained independently associated with COPD severity (OR: 1.701, 95% CI: 1.320–2.191, *P* < 0.001; OR: 1.686, 95% CI: 1.282–2.218, *P* < 0.001). Specifically, patients with CVD had a 1.701 times higher risk of developing severe COPD compared to those without CVD, while patients with hypertension had a 1.686 times higher risk compared to those without hypertension (*P* < 0.05).

**Table 2:** Univariate logistic regression analysis of COPD severity.

	OR	95% CI	*P* value
Age	1.021	1.016–1.025	<0.001
Male	1.342	1.184–1.521	<0.001
Smoking	1.930	1.352–2.755	<0.001
Smoking frequency			0.027
Never smoker	Ref		
Occasional smoker	1.460	0.854–2.494	0.166
Daily smoker	0.695	0.509–0.949	0.022
Chronic bronchitis	1.323	1.006–1.739	0.045
Bronchiectasis	0.570	0.238–1.367	0.208
Emphysema	1.659	1.274–2.161	<0.001
Tuberculosis	1.564	0.789–3.100	0.200
Lung cancer	1.469	0.296–7.285	0.638
Pulmonary heart disease	2.942	0.735–11.771	0.127
Chronic rhinitis	0.333	0.126–0.879	0.026
Diabetes	1.344	0.743–2.434	0.328
CVD	1.505	1.193–1.897	0.001
CHD	1.878	1.072–3.289	0.028
Arrhythmia	1.471	0.660–3.278	0.346
Hypertension	1.383	1.120–1.708	0.003
BMI	0.910	0.896–0.924	<0.001
SBP	0.997	0.988–1.007	0.544
DBP	0.993	0.977–1.009	0.402
Heart rate	1.025	1.009–1.040	0.001
FEV1/FVC	0.833	0.827–0.840	<0.001

COPD, chronic obstructive pulmonary disease; CVD, cardiovascular disease; CHD, coronary heart disease; BMI, body mass index; SBP, systolic blood pressure; DBP, diastolic blood pressure; FEV1, forced expiratory volume in the first second; FVC, forced vital capacity; OR, odds ratio; CI, confidence interval.

**Table 3:** Multivariate logistic regression analysis of CVD and its subcategories with COPD severity.

	Model 1			Model 2			Model 3		
	OR	95% CI	*P* value	OR	95% CI	*P* value	OR	95% CI	*P* value
CVD	1.465	1.161–1.849	0.001	1.746	1.367–2.229	<0.001	1.701	1.320–2.191	<0.001
CHD	1.719	0.980–3.018	0.059	2.022	1.146–3.566	0.015	1.772	0.989–3.177	0.055
Arrhythmia	1.441	0.644–3.224	0.374	1.546	0.687–3.480	0.292	1.255	0.536–2.934	0.601
Hypertension	1.367	1.106–1.691	0.004	1.608	1.283–2.015	<0.001	1.686	1.282–2.218	<0.001

Model 1: Adjusted for age and gender; Model 2: Adjusted for age, gender, smoking frequency, and BMI; Model 3: Adjusted for age, gender, smoking frequency, BMI, chronic bronchitis, chronic rhinitis, and emphysema. CVD, cardiovascular disease; COPD, chronic obstructive pulmonary disease; CHD, coronary heart disease; BMI, body mass index; OR, odds ratio; CI, confidence interval.

### Stratified analysis

Subgroup analysis revealed an independent association between CVD and COPD severity in males, individuals aged ≤ 70 years, those aged > 70 years, individuals with a BMI < 24 or BMI ≥ 24 kg/m², and never smokers (*P* < 0.05). Additionally, CHD showed a significant association with COPD severity in individuals aged ≤ 70 years and never smokers, while hypertension was associated with COPD severity in males, individuals aged ≤ 70 years, those aged > 70 years, individuals with a BMI < 24 or BMI ≥ 24 kg/m², and never smokers (*P* < 0.05) (Table 4).

**Table 4:** Multivariable stratified analysis of the association between CVD, subtypes, and COPD severity.

	CVD			CHD			Hypertension		
	OR	95% CI	*P* value	OR	95% CI	*P* value	OR	95% CI	*P* value
Gender									
Male	1.898	1.432–2.516	<0.001	1.741	0.926–3.270	0.085	1.902	1.401–2.584	<0.001
Female	0.980	0.515–1.866	0.952	2.286	0.472–11.079	0.304	0.910	0.455–1.819	0.789
Age, years									
≤70	1.983	1.408–2.792	<0.001	2.950	1.157–7.525	0.024	1.883	1.302–2.724	<0.001
>70	1.498	1.204–2.191	0.037	1.366	0.645–2.893	0.415	1.563	1.035–2.361	0.034
BMI, kg/m^2^									
<24	1.360	1.006–1.837	0.046	1.289	0.614–2.709	0.503	1.420	1.007–2.004	0.046
≥24	1.886	1.222–2.911	0.004	2.489	0.966–6.415	0.059	1.786	1.137–2.805	0.012
Smoking frequency									
Never smoker	1.956	1.473–2.597	<0.001	2.199	1.132–4.270	0.020	1.948	1.431–2.653	<0.001
Occasional smoker	1.176	0.254–5.395	0.835	0.611	0.029–12.782	0.751	1.562	0.298–8.023	0.598
Daily smoker	1.265	0.616–2.600	0.522	1.589	0.343–7.357	0.554	1.238	0.592–2.591	0.571

The multivariable regression model was adjusted for age, gender, smoking frequency, BMI, chronic bronchitis, chronic rhinitis, and emphysema, except for the grouping variables. CVD, cardiovascular disease; COPD, chronic obstructive pulmonary disease; CHD, coronary heart disease; BMI, body mass index; OR, odds ratio; CI, confidence interval.

### Sensitivity analysis

Sensitivity analysis showed (Table 5), CVD and hypertension remained independently associated with COPD severity after excluding people with bronchiectasis, tuberculosis, lung cancer, pulmonary heart disease, and diabetes (*P* < 0.05). Specifically, the risk of severe COPD was 1.656 times higher in patients with CVD than in patients without CVD (95% CI: 1.254–2.186, *P* < 0.001), whereas the risk of severe COPD was 1.706 times higher in patients with hypertension than in patients without hypertension (95% CI: 1.266–2.229, *P* < 0.001).

**Table 5:** Multivariate logistic regression analysis of the association between CVD, subtypes, and COPD severity: excluding bronchiectasis, tuberculosis, lung cancer, pulmonary heart disease, and diabetes.

	Model 1			Model 2			Model 3		
	OR	95% CI	*P* value	OR	95% CI	*P* value	OR	95% CI	*P* value
CVD	1.410	1.093–1.819	0.008	1.521	1.167–1.982	0.002	1.656	1.254–2.186	<0.001
CHD	1.619	0.878–2.986	0.123	1.694	0.913–3.142	0.095	1.698	0.869–3.218	0.105
Arrhythmia	1.200	0.471–3.059	0.702	1.192	0.467–3.043	0.713	0.932	0.347–2.501	0.889
Hypertension	1.419	1.078–1.869	0.013	1.532	1.152–2.037	0.003	1.706	1.266–2.299	<0.001

Model 1: Adjusted for age and gender; Model 2: Adjusted for age, gender, smoking frequency, and BMI; Model 3: Adjusted for age, gender, smoking frequency, BMI, chronic bronchitis, chronic rhinitis, and emphysema. CVD, cardiovascular disease; COPD, chronic obstructive pulmonary disease; CHD, coronary heart disease; BMI, body mass index; OR, odds ratio; CI, confidence interval.

## Discussion

In this study, we investigated the association between CVD and their subtypes with the severity of COPD. Our results demonstrated that CVD, particularly hypertension, was consistently and significantly associated with COPD severity, even after adjusting for multiple confounders. Although CHD did not show statistically significant differences between univariate and multivariate analyses, stratified analyses revealed that individuals with CHD had more severe COPD, particularly those aged ≤ 70 years and never smokers. These findings have important clinical implications for the early detection and prevention of severe COPD.

Current evidence suggests that CVD is associated with COPD. For example, Bai et al. found that patients at higher cardiovascular risk had a more rapid decline in lung function, especially for FEV1 and FVC, and that the rate of decline was further accelerated in those with CVD. High levels of physical and social activity can mitigate the negative effects of CVD, thus highlighting that we can intervene to reduce the impact of CVD on lung function (18). Secondly, Alter et al. found that CHD and hypertension were associated with all-cause mortality in COPD. Chronic coronary artery disease and hypertension are associated with increased mortality in patients with COPD, which underscores the need for extensive diagnostic testing in patients with COPD, especially to assess cardiovascular comorbidities (19). In contrast, Chen et al. found that patients with COPD had a higher prevalence of one or more CVD (including CHD, heart failure, heart attack and diabetes) compared to patients without COPD, which highlights the importance of the prevention and management of CVD in patients with COPD (5). In addition, Dalal et al. found that patients with COPD had a higher prevalence of CVD, which in turn led to an increased risk of mortality from cardiovascular-related causes. CVD as a co-morbidity may include several forms of disease such as angina, arrhythmia, cardiac hypertrophy and myocardial infarction. The presence of these CVD severely affects the survival expectancy of patients with COPD, significantly reducing their quality of life and the likelihood of long-term survival. which suggests a close association between COPD and CVD, and further studies are needed to explore the association (20). Almagro et al. discovered a close relationship between COPD and CVD, noting that the treatment strategies for these two conditions influence each other. This highlights the critical importance of appropriately managing both diseases (21). Furthermore, a Canadian COPD cohort study demonstrated a significant association between impaired lung function and the prevalence and incidence of CVD, particularly in patients with moderate to severe COPD. The study found that, compared with individuals with normal lung function, the prevalence of CVD was significantly higher in those with impaired lung function, with OR of 1.66 for the impaired lung function group and 1.55 for the COPD group. The hazard ratio for CVD incidence in both groups was approximately 2.09. Although adding impaired lung function to existing risk scores only slightly improved their predictive ability, the study emphasized the importance of early identification and intervention in individuals with impaired lung function to reduce CVD risk (22). In contrast, this study, based on a large cross-sectional cohort of COPD patients from Chinese hospitals, further explores the relationship between CVD and its subcategories (such as hypertension) with the severity of COPD. Both studies revealed a strong link between CVD and COPD; however, this study, through multivariable regression analysis, subgroup analysis, and sensitivity analysis, found that CVD and hypertension increased the risk of developing severe or very severe COPD by 1.701 times and 1.686 times, respectively. This association was particularly stable among men, individuals aged ≤ 70 years, those with different BMI categories, and never smokers. Sensitivity analyses, excluding potential confounding conditions such as bronchiectasis, tuberculosis, and pulmonary heart disease, confirmed the robustness of these findings. Additionally, unlike the longitudinal design of the Canadian cohort study, this study employed a cross-sectional design. While the cross-sectional approach revealed a strong association between CVD and COPD severity, it could not establish a causal relationship, highlighting the need for future prospective studies to validate these findings. In summary, both studies underscore the critical impact of CVD on COPD patients. The Canadian cohort study focuses on the risk of developing CVD, while this study emphasizes the relationship between CVD and the severity of COPD and its clinical management implications, reflecting the complementary focus of these investigations.

Despite the exciting findings of this study, the underlying biological mechanisms remain unknown. After conducting literature research, we identified several possible mechanisms between CVD and COPD. Firstly, traditional cardiovascular risk factors such as age and insulin resistance may also contribute to the accumulation of lipids between the ribs, and furthermore lead to a decrease in pulmonary static elastic recoil, which reduces lung function and ultimately exacerbates the condition of COPD patients (23, 24). Secondly, there may also be common pathogenic mechanisms between both CVD and COPD, such as inflammation, oxidative stress, and programmed cell death (25–28). Inflammation and oxidative stress play an important role in the occurrence and development of COPD. Inflammation is considered to be one of the causes of coronary atherosclerosis, especially in the case of coronary atherosclerosis, which includes blood-derived immune and inflammatory cells. Meanwhile, reactive oxygen species have also been shown to promote the formation of atherosclerotic plaques (29). Smoking, in particular, not only increases the level of inflammation in the body, but also leads to the formation and rupture of atherosclerotic plaques as well as an increased risk of CVD, which is more closely related to the development of COPD (30). These common associates may act on each other and exacerbate COPD in patients with CVD. Third, inflammation not only promotes the development of atherosclerosis by promoting the growth and migration of vascular smooth muscle cells, but also the damage of small airways and the loss of lung parenchyma. Since atherosclerosis and small airway lesions are the basis of CVD and COPD pathology, respectively, inflammation may be a potential biological mechanism linking the association of CVD and COPD (31). However, there are still more potential mechanisms that need to be further explored by more cellular and animal experiments.

In this study, we also provided some advantages for the clinical management of COPD. For example, this study was conducted using a standardised collection of subject information, which collected information from a representative sample of the Chinese population with a large and representative sample size. Secondly, in the baseline collection, we collected patients' lung function and grouped the subjects according to the standardised method, which explored the association between CVD and severity of COPD in a deeper and multidimensional way, and provided some clinical value and practical guidance for the clinical monitoring and management of COPD patients. However, this study also had certain flaws. For example, in this study, we only conducted a cross-sectional study, so we could only verify the association between CVD and COPD severity, but not the causal relationship, so we need to improve the relevant studies to continue to explore the relationship between the two. Secondly, the information of patients' CVD and its subclassification was collected from questionnaires without on-site monitoring and assessment by medical professionals, which had some recall bias, so more perfect prospective clinical trials are needed to further verify the relationship between the two. In addition, this study only focused on the Chinese population, which may limit the application of the findings to other countries and ethnicities. Finally, the current study lacked some common biomarkers (such as C-reactive protein and N-terminal pro-brain natriuretic peptide) to exclude more confounding factors, so more refined and systematic studies are needed to verify the association between the two. Despite these limitations, our study still provides some clues and theoretical basis for CVD in the clinical management of COPD patients and contributes to the promotion of the inclusion of CVD in the future clinical routine management of COPD.

## Conclusion

Patients with severe-very severe COPD are associated with CVD, especially hypertension, compared with patients with mild-to-moderate COPD, which underscores the urgent need for targeted CVD prevention and management strategies in COPD patients. For future research, longitudinal studies are essential to explore the temporal relationship between COPD severity and the development of CVD, particularly hypertension. Interventional studies focusing on pharmacological or lifestyle-based interventions for hypertension in COPD patients should be prioritized. Additionally, more detailed mechanistic studies are needed to understand the pathways linking COPD with cardiovascular outcomes, to inform the development of more precise treatment protocols.

## Data Availability

The datasets used in this study are not publicly available, as our research group is currently working on ongoing projects, and additional related publications are expected. However, the data can be provided upon reasonable request from the corresponding author. Please feel free to reach out if you would like to discuss further.
